# Development and validation of a prognostic model for predicting post-discharge mortality risk in patients with ST-segment elevation myocardial infarction (STEMI) undergoing primary percutaneous coronary intervention (PPCI)

**DOI:** 10.1186/s13019-024-02665-3

**Published:** 2024-03-30

**Authors:** Lingling Zhang, Zhican Liu, Yunlong Zhu, Mingxin Wu, Haobo Huang, Wenbin Yang, Ke Peng, Jianping Zeng

**Affiliations:** 1 Department of Cardiology, Xiangtan Central Hospital, Xiangtan, 411100 China; 2 Graduate Collaborative Training Base of Xiangtan Central Hospital, Hengyang Medical School, University of South China, Hengyang, Hunan 421001 China; 3 Department of Cardiology, the Second Xiangya Hospital of Central South University, Changsha, Hunan 410011 China; 4 Department of Scientific Research, Xiangtan Central Hospital, Xiangtan, 411100 China; 5 Medical Department, Xiangtan Central Hospital, Xiangtan, 411100 China

**Keywords:** ST-segment elevation myocardial infarction (STEMI), All-cause mortality risk, Predictive model, Least Absolute Shrinkage and Selection Operator (LASSO), Decision Curve Analysis (DCA)

## Abstract

**Background:**

Accurately predicting post-discharge mortality risk in patients with ST-segment elevation myocardial infarction (STEMI) undergoing primary percutaneous coronary intervention (PPCI) remains a complex and critical challenge. The primary objective of this study was to develop and validate a robust risk prediction model to assess the 12-month and 24-month mortality risk in STEMI patients after hospital discharge.

**Methods:**

A retrospective study was conducted on 664 STEMI patients who underwent PPCI at Xiangtan Central Hospital Chest Pain Center between 2020 and 2022. The dataset was randomly divided into a training cohort (*n* = 464) and a validation cohort (*n* = 200) using a 7:3 ratio. The primary outcome was all-cause mortality following hospital discharge. The least absolute shrinkage and selection operator (LASSO) regression model was employed to identify the optimal predictive variables. Based on these variables, a regression model was constructed to determine the significant predictors of mortality. The performance of the model was evaluated using receiver operating characteristic (ROC) curve analysis and decision curve analysis (DCA).

**Results:**

The prognostic model was developed based on the LASSO regression results and further validated using the independent validation cohort. LASSO regression identified five important predictors: age, Killip classification, B-type natriuretic peptide precursor (NTpro-BNP), left ventricular ejection fraction (LVEF), and the usage of angiotensin-converting enzyme inhibitors/angiotensin receptor blockers/angiotensin receptor-neprilysin inhibitors (ACEI/ARB/ARNI). The Harrell's concordance index (C-index) for the training and validation cohorts were 0.863 (95% CI: 0.792–0.934) and 0.888 (95% CI: 0.821–0.955), respectively. The area under the curve (AUC) for the training cohort at 12 months and 24 months was 0.785 (95% CI: 0.771–0.948) and 0.812 (95% CI: 0.772–0.940), respectively, while the corresponding values for the validation cohort were 0.864 (95% CI: 0.604–0.965) and 0.845 (95% CI: 0.705–0.951). These results confirm the stability and predictive accuracy of our model, demonstrating its reliable discriminative ability for post-discharge all-cause mortality risk. DCA analysis exhibited favorable net benefit of the nomogram.

**Conclusion:**

The developed nomogram shows potential as a tool for predicting post-discharge mortality in STEMI patients undergoing PPCI. However, its full utility awaits confirmation through broader external and temporal validation.

**Supplementary Information:**

The online version contains supplementary material available at 10.1186/s13019-024-02665-3.

## Introduction

In light of the escalating global prevalence of coronary artery disease, ST-segment elevation myocardial infarction (STEMI) has been identified as a predominant contributor to cardiovascular mortality [1]. While recent advancements in primary percutaneous coronary intervention (PPCI) have significantly improved short-term therapeutic outcomes for STEMI patients, a pronounced long-term mortality risk remains post-discharge [2]. Therefore, the need for detailed risk stratification for these patients is paramount, guiding therapeutic interventions and optimizing long-term clinical outcomes [3, 4].

Numerous evaluative instruments and risk-assessment algorithms have been developed to gauge post-discharge mortality risk among STEMI cohorts [5–8]. However, many of these models draw upon data from extensive clinical trials primarily conducted in European and North American populations, raising questions regarding their applicability and precision for patients outside these regions. Moreover, a majority of these models lean towards conventional statistical methods for variable selection and model development, potentially overlooking crucial predictive factors.

Emerging prominently in both statistical and machine learning disciplines, the Least Absolute Shrinkage and Selection Operator (LASSO) regression technique has positioned itself as an effective tool for feature selection and data dimensionality reduction [9]. By strategically penalizing regression coefficients, LASSO adeptly identifies variables closely associated with prognostic outcomes, leading to a model that is both concise and rigorous [10]. With this perspective, this study aims to employ the LASSO regression approach to develop and validate a new predictive algorithm for post-discharge mortality risk in STEMI patients, using data gathered from the Chest Pain Center at Xiangtan Central Hospital over a three-year period. We anticipate that this novel model will enhance clinical decision-making, refine treatment approaches, and improve long-term survival rates for patients.

## Methods

### Study design and participants

**Fig. 1:**
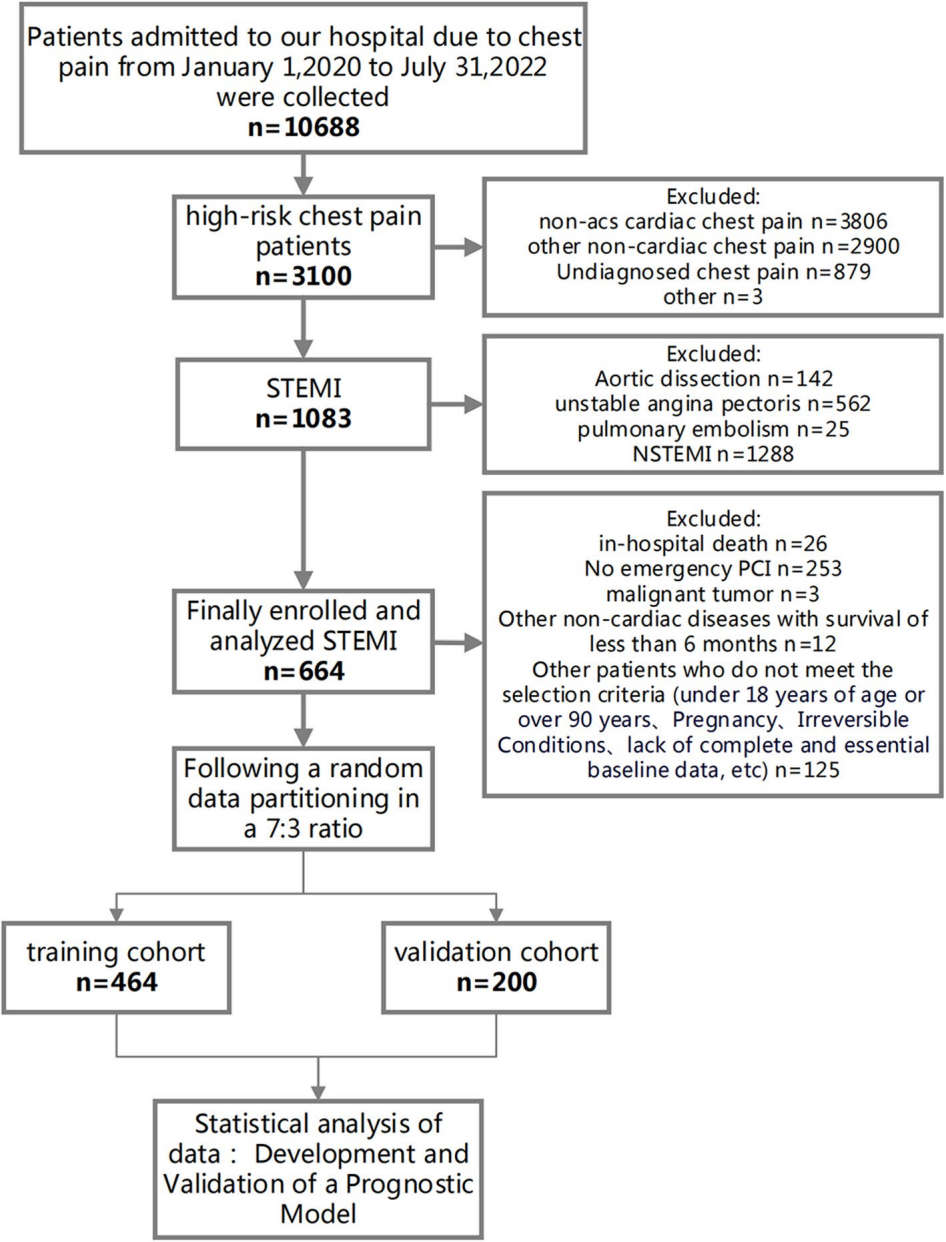
Flow diagram for participant screening, eligibility, and analysis. (Note: The flow diagram in Fig. 1 outlines the process of participant screening, eligibility assessment, and data analysis in the study. The diagram illustrates the sequential steps followed from the initial screening of participants to the final analysis of the collected data)

In this retrospective cohort study, we enrolled 664 ST-segment elevation myocardial infarction (STEMI) patients who underwent percutaneous coronary intervention (PCI) at Xiangtan Central Hospital Chest Pain Center between January 1, 2020, and July 31, 2022 (Fig. 1). The inclusion criteria were: 1) first-time STEMI patients as defined by the guidelines [2]; 2) receipt of emergency PCI treatment. Exclusion criteria included: 1) age under 18 years; 2) missing essential data; 3) in-hospital death; 4) STEMI patients who did not undergo PCI; 5) expected survival of fewer than six months due to malignant tumors or other non-cardiac diseases. The dataset was randomly divided in a 7:3 ratio into training (*n* = 464) and validation (*n* = 200) cohorts.

### Data collection and variable definitions

Patient records were retrieved from the hospital's electronic medical record system and the China Chest Pain Center (CCPC) Data platform. These records comprised demographic information, pre-admission medical history, biochemical indicators upon admission, medication usage, and PCI treatment-related details.

A 'current smoker' is defined as an individual who has regularly smoked tobacco within the past year. Regular smoking is characterized as smoking at least once per week, regardless of the quantity smoked.In parallel, a 'current drinker' is defined as an individual who, over the past year, has consumed an average of at least a certain number of alcohol units per week. One alcohol unit is equivalent to approximately 10 ml or 8 g of pure alcohol, roughly corresponding to one bottle of standard strength (5%) beer, a small glass (125 ml) of wine, or a small shot (25 ml) of spirits.

Cardiogenic shock was defined as a state of critical end-organ hypoperfusion due to primary cardiac dysfunction. This was clinically diagnosed based on a combination of hemodynamic parameters and clinical signs, including sustained hypotension (systolic blood pressure < 90 mmHg for at least 30 min or the need for supportive measures to maintain systolic blood pressure above 90 mmHg), evidence of pulmonary congestion, and signs of impaired organ perfusion.

### Follow-up and outcome measures

We followed up with all study participants until January 31, 2023. A dedicated team of five experienced cardiovascular physicians and two nurses collected outcome events through outpatient visits, telephone follow-ups, and community check-ins. The primary follow-up end point event was the all-cause death risk.

#### Ethics and informed consent

This study was approved by the Ethics Committee of Xiangtan Central Hospital (Xiangtan, China) (Ethics Approval No. 2023–02-001) and adhered to the principles outlined in the Helsinki Declaration. As a retrospective study that only collected clinical data without intervening in patient treatment, informed consent was waived.

### Statistical analysis

All data was normalized through z-score transformation, resulting in a mean of 0 and a standard deviation of 1. For the selection of predictors, we deployed the Least Absolute Shrinkage and Selection Operator (LASSO) regression technique to identify variables exhibiting a significant correlation with all-cause mortality. A regression model was subsequently developed incorporating these selected variables using the glmnet package in R for LASSO regression modeling. Each patient's mortality risk score was computed through a linear combination of the chosen predictive variables and their respective coefficients. The optimal lambda (λ) parameter, minimizing cross-validation error, was selected. The model was refitted using the selected λ and all available observations, causing most covariate coefficients to shrink to zero while retaining only those non-zero coefficients identified by the LASSO procedure. These non-zero coefficients were classified as mortality risk predictors. A mortality risk prediction nomogram was then constructed using the "rms" package. Model performance was assessed via discrimination and calibration analyses. Discrimination was quantified using the area under the Receiver Operating Characteristic (ROC) curve, while calibration was assessed by examining calibration plots. We utilized Decision Curve Analysis (DCA) to assess the clinical utility of our predictive model. DCA quantifies the net benefits of a model at various threshold probabilities, balancing the true positives against the false positives [11]. This approach helps in identifying clinically relevant threshold ranges where the model provides significant decision-making advantages. The predictive accuracy of the risk model was evaluated via the C-statistics for discrimination and the Hosmer–Lemeshow chi-square test for calibration.

Group differences were assessed using independent samples t-tests, chi-square tests, or Mann–Whitney U tests as appropriate. Normality was tested with the Kolmogorov–Smirnov test. Normally distributed continuous variables were reported as mean ± standard deviation, while non-normally distributed continuous variables were expressed as medians with interquartile ranges. Categorical variables were presented as n (%). All statistical tests were two-sided, with a *p*-value of < 0.05 deemed statistically significant. Model development, discrimination, and calibration performance were evaluated using similar methods. All statistical analyses were conducted using R software, version 4.2.0 (http://www.R-project.org).

## Results

Table 1 delineates the baseline characteristics of patients with STEMI following PPCI intervention, categorized into the training cohort (*N* = 464) and the validation cohort (*N* = 200). The male constituent in both cohorts was 78.2% and 76.0%, respectively, with a *P*-value of 0.595. The mean age was reported at 63.0 years; the age distribution for the training cohort ranged between 55.0 and 71.0 years, while the validation cohort exhibited a similar range from 54.0 to 71.0 years (*P* = 0.855). Concerning historical medical data, both the training and validation cohorts demonstrated smoking prevalences of 56.9% and 57.0%, respectively. Therapeutically, β-blocker administration was observed in 88.8% of the training cohort and 89.0% of the validation cohort. In the context of PPCI procedural specifics, the Radial artery technique was the preferred method, with adoption rates of 93.3% in the training cohort and 92.5% in the validation cohort. Mortality indices for the training and validation cohorts were 5.82% and 4.00%, respectively, yielding a *P*-value of 0.439. A comprehensive data set, including statistical figures, *P*-values, and relevant terminologies, is tabulated in Table 1.

**Table 1:** Baseline characteristics of STEMI patients undergoing ppci in the mortality risk prognostic model development and validation

	Survive *N* = 629	Death *N* = 35	*P*-value
**Demographics**			
Male, N (%)	493 (78.38%)	22 (62.86%)	0.032
Age, years	61.92 ± 12.04	69.66 ± 10.58	< 0.001
Obesity	180 (28.62%)	4 (11.43%)	0.027
**Medical history, N (%)**			
Current smoker	365 (58.03%)	13 (37.14%)	0.015
Current drinker	93 (14.79%)	33 (16.5%)	0.136
Stroke	77 (12.24%)	9 (25.71%)	0.021
Diabetes	175 (27.82%)	11 (31.43%)	0.644
Renal insufficiency	85 (13.51%)	13 (37.14%)	< 0.001
Hypertension	357 (56.76%)	25 (71.43%)	0.087
Hyperlipidemia	244 (38.79%)	9 (25.71%)	0.121
Atrial fibrillation	44 (7.00%)	8 (22.86%)	< 0.001
Heart valve disease	95 (15.10%)	8 (22.86%)	0.217
Cardiomyopathy	23 (3.66%)	3 (8.57%)	0.145
Anemia	110 (17.49%)	5 (14.29%)	0.626
**Clinical conditions at admission**			
**Killip Classification**			< 0.001
1	394 (62.64%)	8 (22.86%)	
2	149 (23.69%)	9 (25.71%)	
3	14 (2.23%)	1 (2.86%)	
4	72 (11.45%)	17 (48.57%)	
NT-proBNP/100, pg/ml	15.18 ± 30.18	55.33 ± 80.43	< 0.001
TnT, ng/mL	4.99 ± 3.57	4.70 ± 3.72	0.643
LVEF, %	51.16 ± 8.70	45.00 ± 9.42	< 0.001
**Treatment, N (%)**			
Beta-blocker	566 (89.98%)	24 (68.57%)	< 0.001
ACEI/ARB/ARNI	548 (87.12%)	19 (54.29%)	< 0.001
Diuretics	349 (55.48%)	20 (57.14%)	0.848
**PPCI related situation**			
**Surgical approach**			0.002
Radial artery	590 (93.80%)	28 (80.00%)	
Femoral artery	39 (6.20%)	7 (20.00%)	
**Main diseased vessel**			0.017
LAD	294 (46.74%)	17 (48.57%)	
LCX	61 (9.70%)	4 (11.43%)	
RCA	270 (42.93%)	12 (34.29%)	
LM	4 (0.64%)	2 (5.71%)	
**Stenosis degree**			0.726
90–99%	179 (28.46%)	9 (25.71%)	
100%	450 (71.54%)	26 (74.29%)	
**Preoperative TIMI**			0.838
0	452 (71.86%)	26 (74.29%)	
1	22 (3.50%)	2 (5.71%)	
2	102 (16.22%)	5 (14.29%)	
3	53 (8.43%)	2 (5.71%)	
**Implanted stents, N**			0.423
0	89 (14.15%)	8 (22.86%)	
1	431 (68.52%)	23 (65.71%)	
2	96 (15.26%)	4 (11.43%)	
3	13 (2.07%)	0 (0.00%)	
**Complication**			
Bleeding	13 (2.07%)	0 (0.00%)	0.39
Shock	33 (5.25%)	7 (20.00%)	< 0.001
Infect	98 (15.58%)	5 (14.29%)	0.837
New heart failure in hospital	17 (2.70%)	3 (8.57%)	0.048
**CPC quality control index, min**			
Diagnosis-to-loading dose DAPT	9.28 ± 8.77	12.69 ± 11.35	0.028
D to B	68.72 ± 26.38	84.57 ± 31.79	< 0.001
Total ischemic time	419.06 ± 527.37	433.37 ± 384.58	0.874
PCI informed consent time	11.04 ± 10.02	17.34 ± 17.44	< 0.001
CL activation time	13.68 ± 10.65	16.06 ± 9.20	0.196
Consultation time(notice to arrival)	3.29 ± 1.68	4.11 ± 2.81	0.007
FMC-to-ECG	4.34 ± 3.51	4.46 ± 3.52	0.852

According to 7:3, the population is randomly classified into training cohort and Validation cohort. Categorical variables were presented as n (%). Values for continuous variables are given as means ± SD or medians with interquartile ranges

*Abbreviations*: *STEMI* ST-segment elevation myocardial infarction, *P-value* Probability value, *N/n* number*, NT-proBNP* N-terminal pro-B type natriureti peptide, *TnT* Troponin T, *LVEF* left ventricular ejection fraction, *ACEI* angiotensin-converting enzyme inhibitors, *ARB* angiotensin receptor blockers, *ARNI* angiotensin receptor-enkephalase inhibitors, *PPCI* primary percutaneous coronary intervention, *LAD* left anterior descending artery, *LCX* left circumflex artery, *RCA* right coronary arter, *LM* Left Main, *TIMI* thrombolysis in myocardial infarction, *CPC* chest pain center, *DAPT* dual antiplatelet therapy, *D-to-B* door-to-balloon, CL catheter lab, *FMC* first medical contact, *ECG* electrocardiogram

Table 2 offers an incisive univariate Cox regression analysis elucidating the mortality risk post-discharge in STEMI patients who underwent PPCI intervention. A one-year increment in age emerged as a salient factor, correlating with an amplified mortality risk (HR = 1.062, 95% CI: 1.026–1.099, *P* = 0.001). History of cerebrovascular events, notably stroke, signaled a heightened death risk (HR = 2.45, 95% CI: 1.035–5.8, *P* = 0.042). Paradoxically, current smokers exhibited a relative attenuation in mortality risk (HR = 0.439, 95% CI: 0.201–0.959, *P* = 0.039). Renal compromise underscored a conspicuous escalation in mortality risk (HR = 3.775, 95% CI: 1.751–8.136, *P* = 0.001). The presence of atrial fibrillation corresponded with a marked surge in mortality risk (HR = 2.818, 95% CI: 1.067–7.447, *P* = 0.037). Significantly, mortality risk metrics within the Killip classification groups II-VI superseded that of the Killip classification group I (HR = 2.05, 95% CI: 1.529–2.747, *P* < 0.001). From a biochemical perspective, an increment of 100 units in NT-proBNP subtly paralleled with an augmented mortality risk (HR = 1.009, 95% CI: 1.005–1.013, *P* < 0.001). Every 1% reduction in left ventricular ejection fraction portended an elevated mortality risk (HR = 0.921, 95% CI: 0.887–0.956, *P* < 0.001). Medicinally, the administration of Beta-blockers (HR = 0.275, 95% CI: 0.12–0.629, *P* = 0.002) and agents from the ACEI/ARB/ARNI spectrum (HR = 0.16, 95% CI: 0.075–0.342, *P* < 0.001) resonated with a conspicuous decrement in mortality risk. The onset of hemodynamic shock was identified as a pivotal exacerbator of mortality risk (HR = 3.655, 95% CI: 1.384–9.657, *P* = 0.009). A more granular inspection of the data can be ascertained in Table 2.

**Table 2:** Univariate cox regression analysis of factors associated with mortality risk in STEMI patients undergoing PPCI

Characteristics	HR	CI%	Z-score	*P-value*
Male vs Female	0.549	0.247–1.223	-1.468	0.142
Age, per year	1.062	1.026–1.099	3.411	**0.001**
Obesity	0.385	0.133–1.114	-1.76	0.078
Current smoker	0.439	0.201–0.959	-2.064	**0.039**
Current drinker	0.523	0.124–2.209	-0.882	0.378
Stroke	2.45	1.035–5.8	2.038	**0.042**
Diabetes	0.92	0.389–2.175	-0.19	0.849
Renal insufficiency	3.775	1.751–8.136	3.39	**0.001**
Hypertension	1.625	0.73–3.618	1.188	0.235
Hyperlipidemia	0.543	0.229–1.284	-1.391	0.164
Atrial fibrillation	2.818	1.067–7.447	2.09	**0.037**
Heart valve disease	1.246	0.471–3.294	0.443	0.657
Cardiomyopathy	1.705	0.4–7.268	0.721	0.471
Anemia	0.849	0.293–2.458	-0.302	0.763
Killip Classification, II-VI vs I	2.05	1.529–2.747	4.8	**< 0.001**
NT-proBNP/100	1.009	1.005–1.013	4.816	**< 0.001**
TnT	0.97	0.872–1.079	-0.561	0.575
LVEF	0.921	0.887–0.956	-4.306	**< 0.001**
Beta-blocker	0.275	0.12–0.629	-3.06	**0.002**
ACEI/ARB/ARNI	0.16	0.075–0.342	-4.736	**< 0.001**
Diuretics	0.817	0.384–1.738	-0.525	0.6
Surgical approach, Radial artery vs Femoral artery	2.344	0.81–6.783	1.571	0.116
Main diseased vessel, others vs LAD	0.808	0.537–1.217	-1.02	0.308
Stenosis degree, 100% vs 90–99%	1.034	0.453–2.362	0.079	0.937
Preoperative TIMI, 1–3 vs 0	0.906	0.62–1.326	-0.507	0.612
Implanted stents, 1–3 vs 0	0.619	0.311–1.232	-1.366	0.172
Infect	0.976	0.337–2.826	-0.045	0.964
Shock	3.655	1.384–9.657	2.615	**0.009**
Bleeding	0	0-Inf	-0.004	0.997
New heart failure in hospital	2.605	0.617–11	1.303	0.193

Bold represent significant values (*p* < 0.05)

*Abbreviations*: *STEMI* ST-segment elevation myocardial infarction, *PPCI* primary percutaneous coronary intervention, *P-value* Probability value, *HR* Hazard Ratio, *CI* Confidence Interval, *N/n* number, *NT-proBNP*:N-terminal pro-B type natriureti peptide, *TnT* Troponin T, *LVEF* left ventricular ejection fraction, *ACEI* angiotensin-converting enzyme inhibitors, *ARB* angiotensin receptor blockers, *ARNI* angiotensin receptor-enkephalase inhibitors, *TIMI* thrombolysis in myocardial infarction

**Fig. 2:**
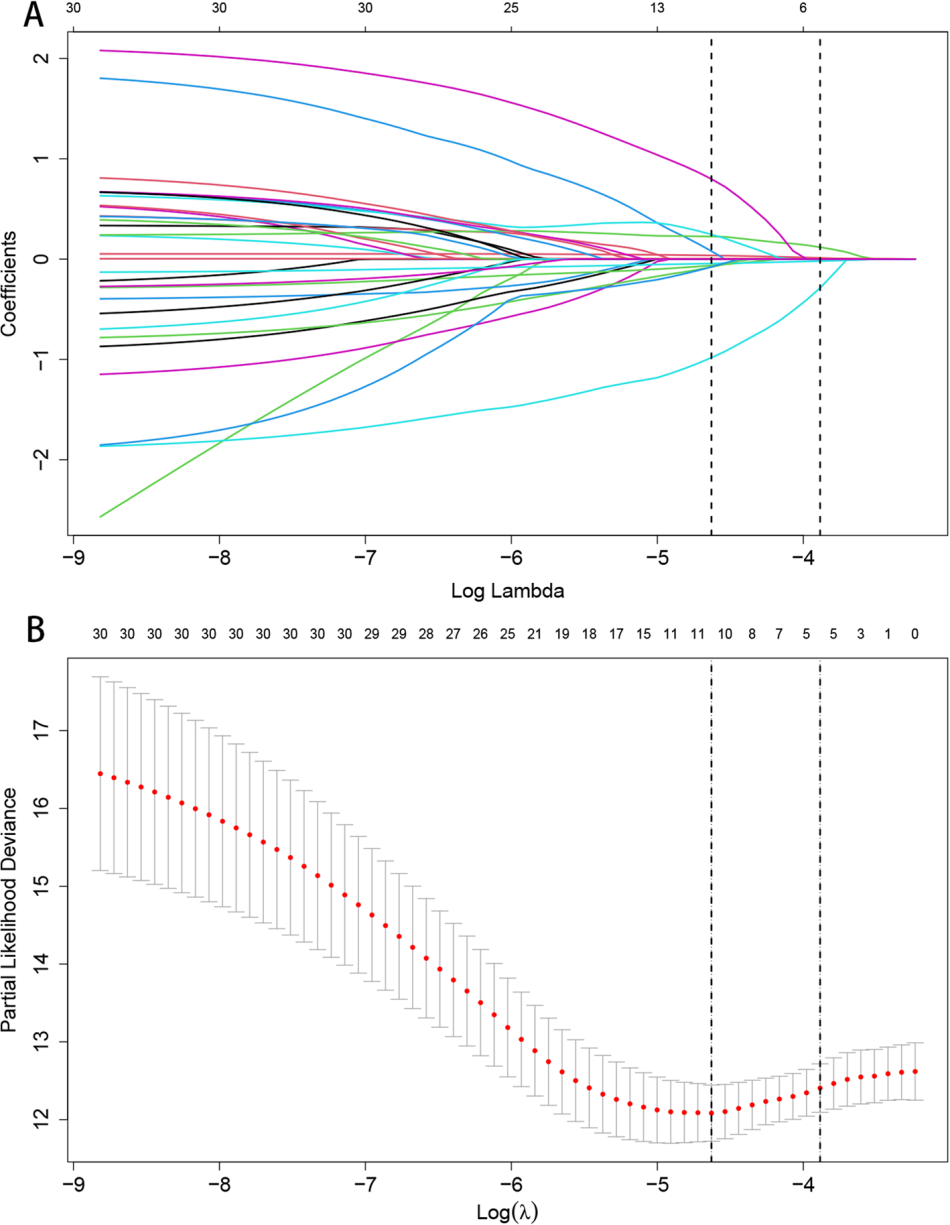
LASSO Regression Coefficient Path and CV LASSO Regression Coefficient Path. **A** LASSO Regression Coefficient Path. **B** CV LASSO Regression Coefficient Path. (Note:The LASSO regression coefficient path displays how the coefficients of each variable change with increasing regularization parameter λ.The CV LASSO regression coefficient path illustrates the coefficients' behavior with λ tuned through cross-validation. Both paths provide insights into the impact of regularization on variable selection and coefficient estimation in the LASSO regression model)

Through the application of the LASSO regression model, we discerned five pivotal prognostic factors robustly correlated with mortality outcomes: age, the Killip classification, NT-proBNP levels, LVEF, and the administration of ACEI/ARB/ARNI therapies, as illustrated in Fig. 2.

Detailed outcomes from the COX multivariable regression analysis, derived from the quintet of predictors elicited by LASSO regression, are tabulated in Table 3:

**Table 3:** Multivariable cox regression analysis of factors associated with mortality risk in STEMI patients undergoing PPCI

Characteristics	β	SE	HR	CI 95%	Z-score	*P-value*
Age, per year	0.046	0.017	1.047	1.012–1.083	2.66	0.008
Killip Classification	0.416	0.166	1.515	1.094–2.098	2.503	0.012
NT-proBNP/100	0.005	0.002	1.005	1.001–1.009	2.008	0.045
LVEF	-0.049	0.023	0.952	0.911–0.995	-2.195	0.028
ACEI/ARB/ARNI	-1.610	0.414	0.200	0.089–0.450	-3.89	< 0.001

Bold represent significant values (*p* < 0.05)

*Abbreviations*: *STEMI* ST-segment elevation myocardial infarction, *PPCI* primary percutaneous coronary intervention, *P-value* Probability value, *SE* Standard Error, *HR* Hazard Ratio, *CI* Confidence Interval, *NT-proBNP* N-terminal pro-B type natriureti peptide, *LVEF* left ventricular ejection fraction, *ACEI* angiotensin-converting enzyme inhibitors, *ARB* angiotensin receptor blockers, *ARNI* angiotensin receptor-enkephalase inhibitors

Incremental age, specified as each advancing year, was linked to a pronounced escalation in mortality risk (HR = 1.047, 95% CI: 1.012–1.083, *P* = 0.008). A heightened Killip classification substantially correlated with augmented mortality risk (HR = 1.515, 95% CI: 1.094–2.098, *P* = 0.012). For every centesimal augmentation in NT-proBNP levels, a discernible amplification in mortality risk was evident (HR = 1.005, 95% CI: 1.001–1.009, *P* = 0.045). In contrast, each percentage point elevation in the left ventricular ejection fraction (LVEF) was significantly allied with a decrement in death risk (HR = 0.952, 95% CI: 0.911–0.995, *P* = 0.028). Notably, patients undergoing ACEI/ARB/ARNI therapeutic regimens manifested a marked diminution in mortality susceptibility (HR = 0.200, 95% CI: 0.089–0.450, *P* < 0.001).

**Fig. 3:**
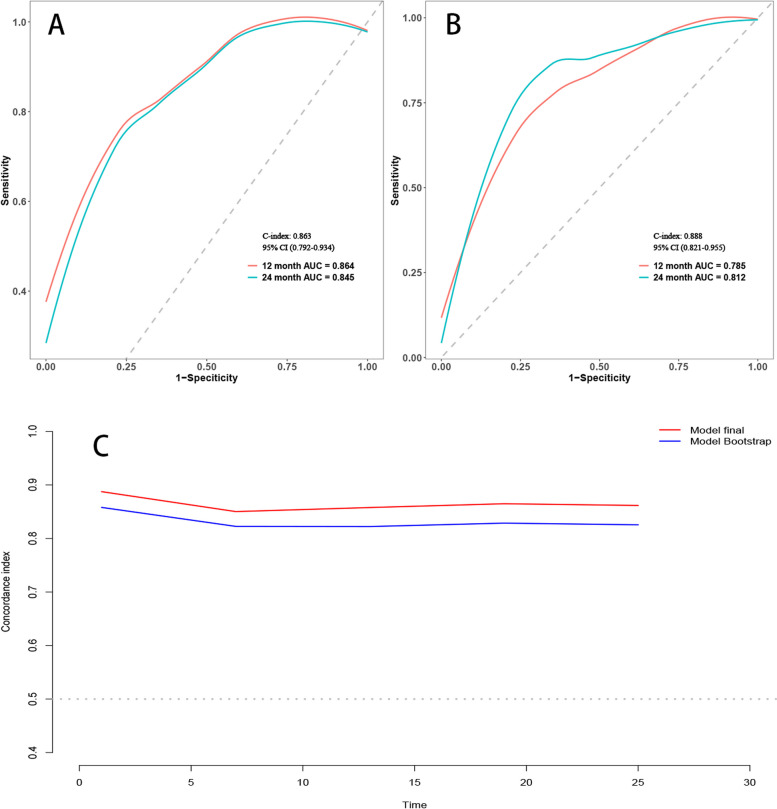
Area under the Receiver Operating Characteristic (ROC) curve and Bootstrap validation. **A** ROC curves for the training set at 12 months and 24 months. **B** ROC curves for the validation set at 12 months and 24 months. **C** Comparison of model stability between the original model and 500 rounds of Bootstrap validation on the training set

**Fig. 4:**
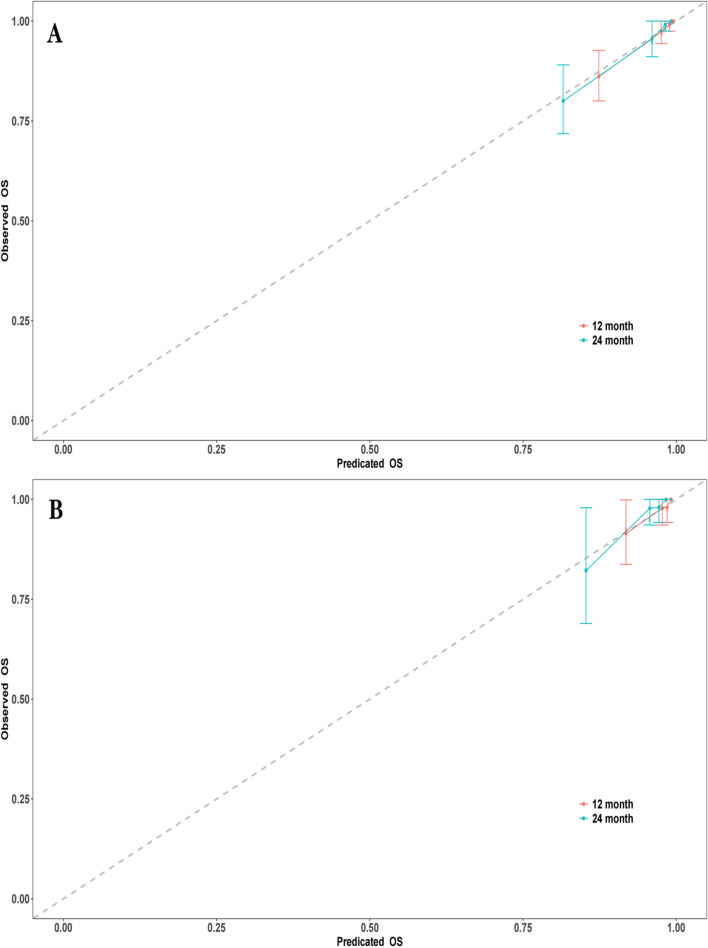
Calibration curves at different time points. **A** Calibration curves for the training set at 12 months and 24 months. **B** Calibration curves for the validation set at 12 months and 24 months. (Note: The calibration curves depict the agreement between the predicted probabilities and the observed outcomes at different time points. The curves represent the performance of the predictive model in terms of calibration, indicating how well the model's predicted probabilities align with the actual probabilities)

Employing time-dependent ROC curves, we elucidated the model's discriminative prowess. A C-index of 0.863 was observed in the training cohort, with a 95% CI ranging from 0.792 to 0.934. This corresponded to AUC values of 0.864 and 0.845 at the 12-month and 24-month intervals, respectively. A C-index of 0.888 was evident for the validation set, enveloped by a 95% CI of 0.821–0.955. This translated to AUC metrics of 0.785 and 0.812 for the 12 and 24 months, respectively (Fig. 3A and B). Post the execution of 500 bootstrap resampling iterations, the model's intrinsic stability was emphatically confirmed (Fig. 3C). Temporal calibration curves ratified impeccable model alignment across the 12- and 24-month benchmarks for the training and validation sets, thereby underscoring the model's robustness (Fig. 4).

**Fig. 5:**
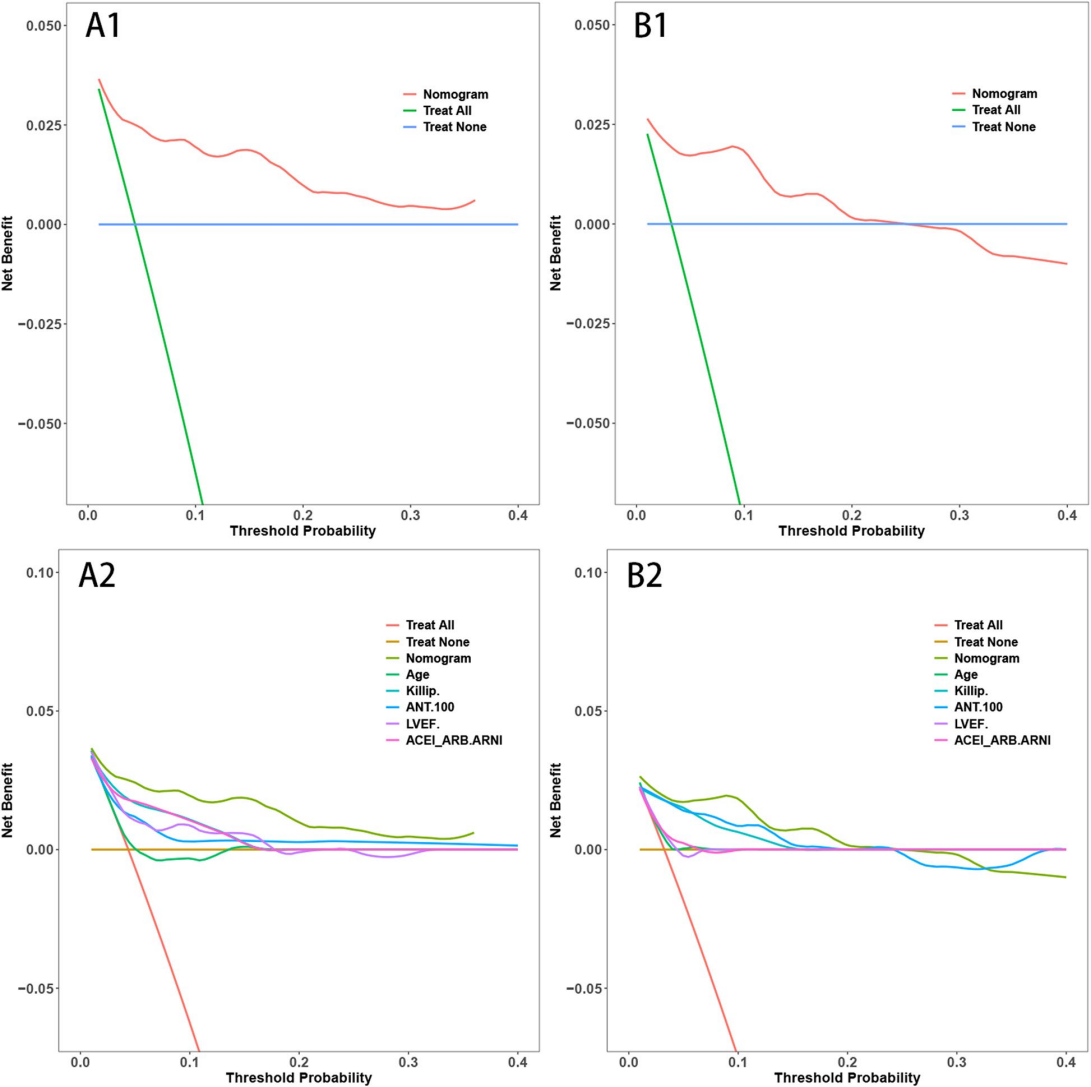
Decision Curve Analysis (DCA) with Time and DCA Nomogram. A1: DCA with Time for the Training Set. B1: DCA with Time for the Validation Set. A2: DCA Nomogram for the Training Set. B2: DCA Nomogram for the Validation Set. (Note: The DCA curves in A1 and B1 illustrate the net benefit of the predictive model over a range of threshold probabilities at different time points for the training and validation sets. These curves provide insights into the clinical usefulness and added value of the model compared to alternative decision strategies. Additionally, the DCA nomograms in A2 and B2 provide a graphical representation of the decision curves, allowing for a more intuitive interpretation and application of the model's results)

**Fig. 6:**
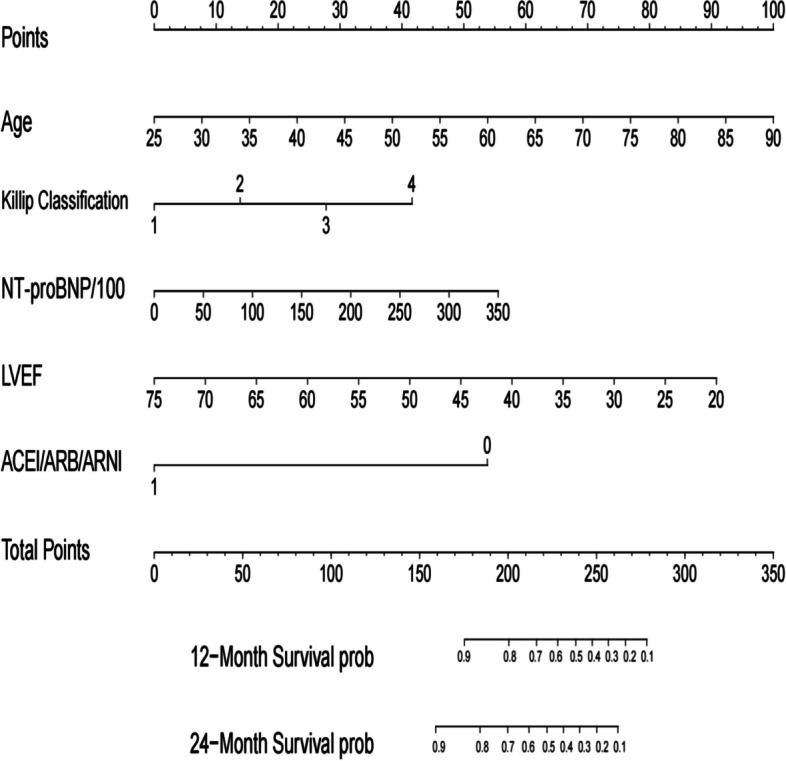
Nomogram for all-cause mortality risk prediction. (Note: The nomogram presents a visual tool for predicting the risk of all-cause mortality. It combines various predictors or risk factors into a comprehensive model that provides an individualized risk assessment. The nomogram allows for a simple and intuitive estimation of the probability of mortality based on the values assigned to each predictor. Clinicians can use this nomogram as a practical aid in risk assessment and shared decision-making with patients regarding appropriate interventions and management strategies)

**Fig. 7:**
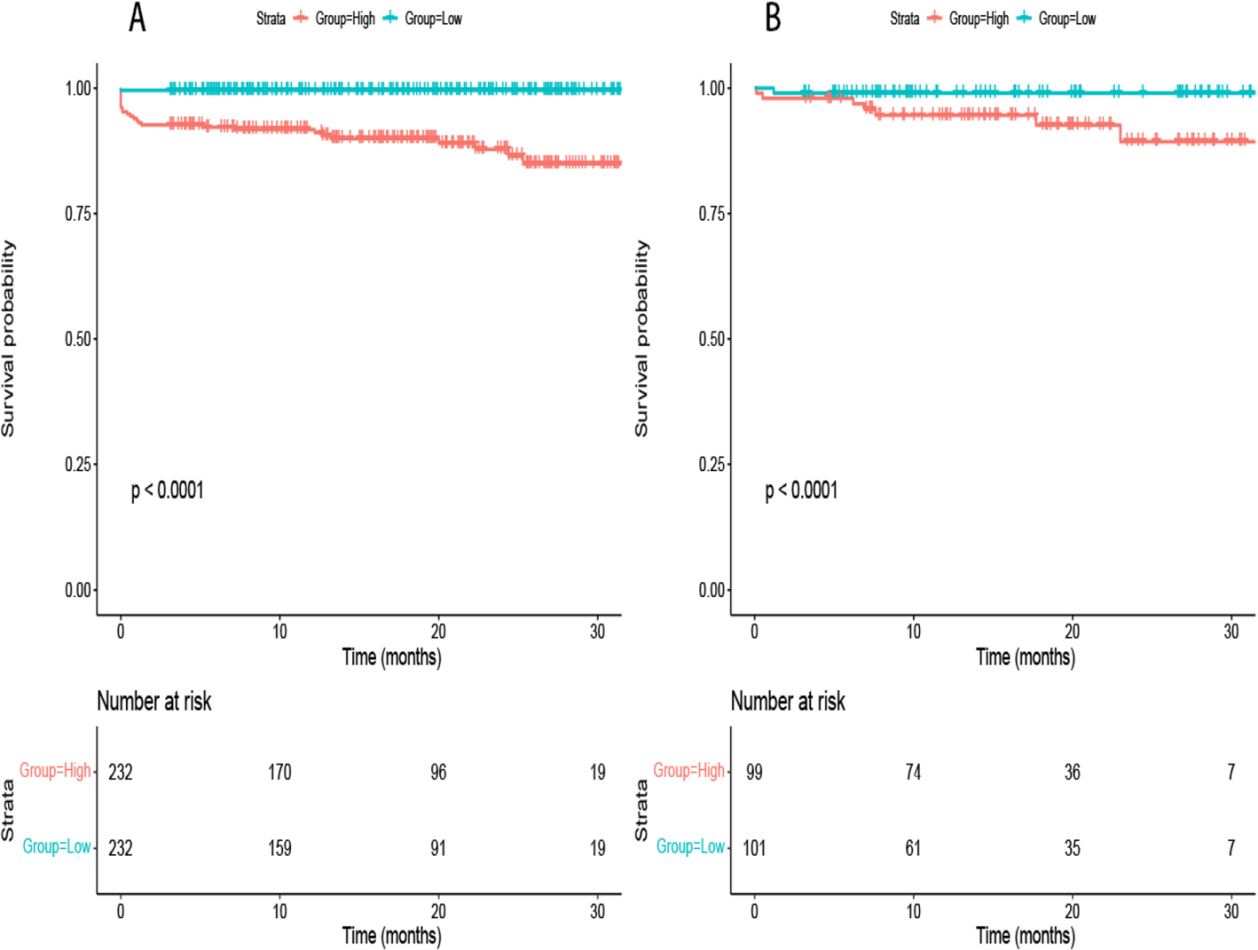
Rationality Analysis: Kaplan–Meier Survival Curves of High-Score and Low-Score Groups. **A** Rationality Analysis for the Training Set. **B** Rationality Analysis for the Validation Set. (Note: The Kaplan–Meier survival curves depicted in **A** and **B** demonstrate the differences in survival outcomes between the high-score and low-score groups. These curves serve as a rationality analysis to evaluate the predictive performance of the scoring system or model. The separation of the survival curves indicates the ability of the scoring system to stratify patients into distinct risk groups. This analysis provides insights into the reliability and validity of the scoring system in predicting survival outcomes and aids in assessing its clinical utility)

The delineated time-dependent DCA and DCA nomogram across both cohorts unequivocally showcased the net clinical benefit, with the nomogram rendition distinctly surpassing the individual performance of the five discrete subsets (Fig. 5). Figure 6 portrays a delineative chart encapsulating the risk scores ascribed to each predictive variable. Elevated scores inherently resonate with an accentuated prospective mortality threat. Leveraging this schematic, patients were accorded scores and stratified into high and low-risk echelons. The Kaplan–Meier survival trajectories were then harnessed to evaluate the congruence across these cohorts. Indubitably, the risk quotient for mortality was attenuated in the low-risk segment compared to its high-risk counterpart across both data partitions (Fig. 7).

In our analysis presented in Supplementary Table 1, we observed a significant reduction in mortality among STEMI patients using ACE inhibitors (ACEI), angiotensin receptor blockers (ARB), or angiotensin receptor-neprilysin inhibitors (ARNI). This effect was evident in both groups classified by left ventricular ejection fraction (LVEF), with hazard ratios indicating a substantial protective effect of these medications on survival.

### Temporal validation

To address the potential for overfitting and to assess the temporal generalizability of our model, a temporal validation was performed. Supplementary Fig. 1 illustrates the receiver operating characteristic (ROC) curves derived from the predictive model. Panel A presents the ROC curves for mortality predictions at 12 and 24 months post-discharge in the training cohort, which included 480 patients from the 2020–2021 dataset. The model demonstrated good predictive ability with an AUC of 0.819 (95% CI: 0.724–0.914) for 12-month mortality and an AUC of 0.836 (95% CI: 0.761–0.911) for 24-month mortality.

Due to the limited follow-up time available for the validation cohort, which consisted of 184 patients from the 2022 dataset, the model's performance was assessed using shorter-term outcomes. Panel B therefore shows the ROC curves for 6-month and 12-month mortality, yielding AUC values of 0.796 (95% CI: 0.603–0.988) and 0.877 (95% CI: 0.642–1.112), respectively. The shortened follow-up period for the validation cohort necessitated the use of these interim time points for model assessment.

### Comparison of two models

Supplementary Fig. 2 compares the predictive accuracy of two models developed via LASSO regression, using ROC curves for the training set (Panel A) and the validation set (Panel B). Model A, defined by the '1se' criterion, demonstrated an AUC of 0.875 in the training set and 0.763 in the validation set, indicating robustness across both datasets with essential predictors: age, Killip classification, ACEI/ARB/ARNI, ntpro-BNP/100, and LVEF. Model B, the 'min' full model, showed comparable AUCs in the training (0.867) and validation (0.765) sets. The performance similarity in both datasets suggests Model A's parsimony is effective for clinical application without compromising predictive ability.

### Model evaluation metrics

Supplementary Table 2 in our manuscript details critical model evaluation metrics on both training and validation sets. Notably, the model shows a strong Area Under the Receiver Operating Characteristic Curve (AUC) with 0.88 on the training set and 0.795 on the validation set, indicating its robust predictive ability. The accuracy rates of 0.909 (training) and 0.85 (validation) further affirm the model's effectiveness. Additionally, Sensitivity and Specificity values demonstrate balanced performance in identifying positive and negative cases. The Positive and Negative Likelihood Ratios (PLR and NLR) along with Predictive Values (PPV and NPV) underscore the model's precision in predicting outcomes. These metrics collectively highlight the model's reliability and potential applicability in practical scenarios.

## Discussion

**Fig. 8:**
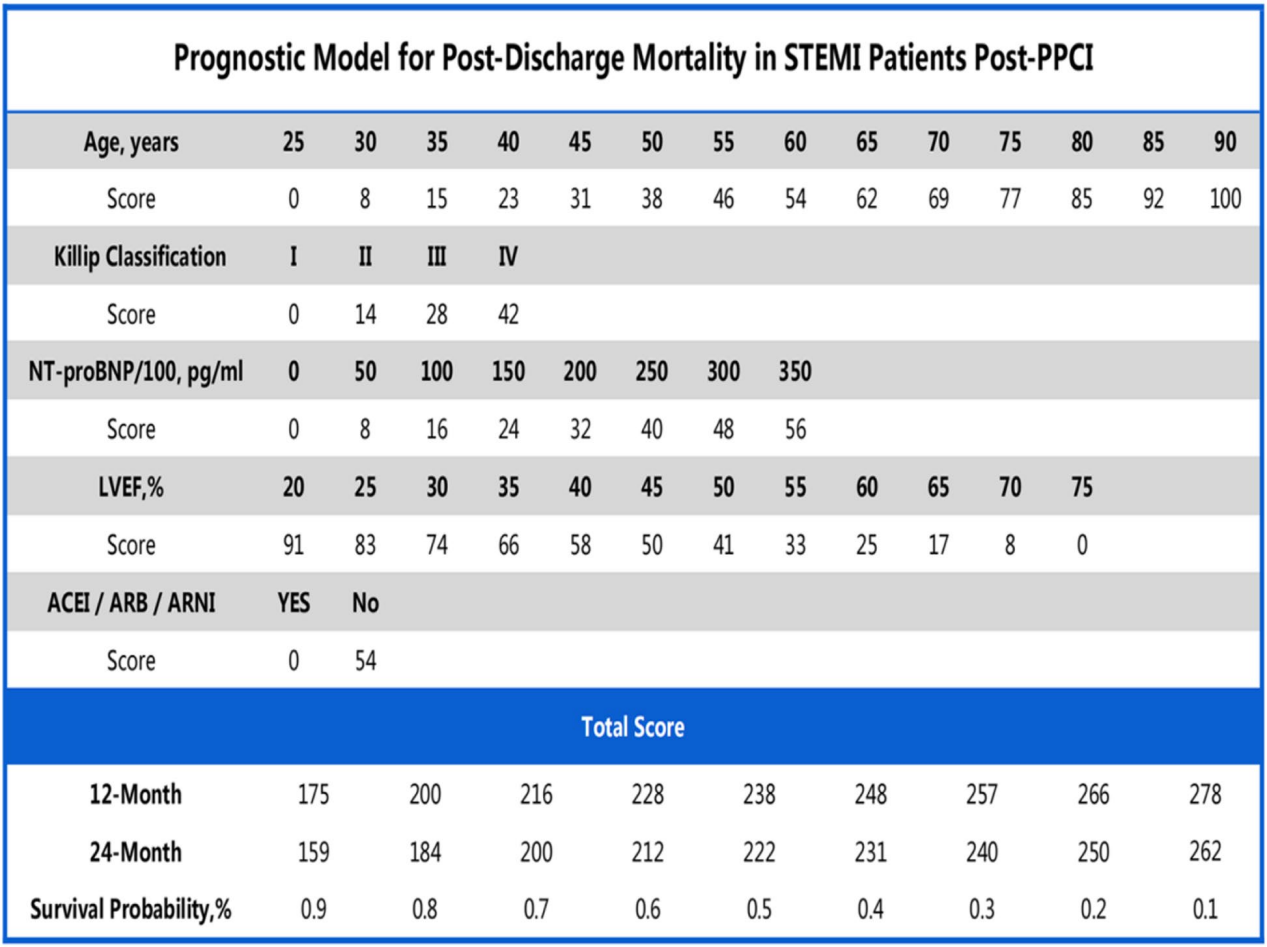
Central Illustration

In the present investigation, we meticulously developed and validated a predictive model that quantifies the 12-month and 24-month post-discharge mortality risks for STEMI patients. The primary predictors integrated into this model include age, the Killip classification, NTpro-BNP concentrations, LVEF values, and the therapeutic use of ACEI/ARB/ARNI (Central Illustration) (Fig. 8). The model's discriminating capability, as evidenced by its C-index and the area under the ROC curve, underscores its reliability and predictive accuracy.

The Killip and Kimball classification has historically been a foundational tool in the early studies of post-STEMI mortality patterns. In their landmark 1967 study, Killip and Kimball thoroughly assessed a cohort of 250 patients and proposed an evaluation framework based on clinical manifestations [12]. This evaluative method remains strongly correlated with mortality outcomes in many contemporary cardiovascular studies despite over five decades. The classification system devised by Killip and Kimball is consistent with findings from recent research [13, 14], highlighting the central role of the Killip classification in prognosticating STEMI outcomes.

In recent years, research centered on NTpro-BNP has gained prominence. In his landmark study, De Lemos delineated a distinct correlation between NTpro-BNP concentrations and the prognostic outcomes of acute coronary syndrome [15]. Our results echo this assertion, endorsing NTpro-BNP as a pivotal prognostic marker for post-STEMI mortality. Contemporary cardiovascular literature further reaffirms the critical role of NTpro-BNP in gauging outcomes among STEMI patients [16, 17].

The therapeutic application of ACEI/ARB remains a cornerstone in the management strategies for myocardial infarction. Pfeffer's pioneering work illuminated the significant role of ACEIs in reducing mortality following myocardial infarction [18]. Our findings align with this perspective, underscoring the beneficial impact of ACEI/ARB in diminishing post-STEMI mortality risks. Beyond Pfeffer's foundational research, recent studies also validate the efficacy of the ACEI/ARB/ARNI ensemble in mitigating cardiovascular event risks [19, 20].

Left Ventricular Ejection Fraction (LVEF) and age are quintessential prognostic indicators for heart failure and coronary artery disease. Solomon's study established a pronounced association between diminishing LVEF and heightened mortality risk [21]. Concurrently, Avezum elucidated that STEMI-associated mortality increases with advancing age [22].

While historical literature consistently underscores the salient roles of age, the Killip classification, and LVEF in stratifying post-myocardial infarction mortality risks, our model introduces a novel integration. It synergistically incorporates these traditional markers with NTpro-BNP concentrations and the therapeutic regimen of ACEI/ARB/ARNI, offering a more encompassing predictive paradigm. Including ACEI/ARB/ARNI in our model reveals a marked reduction in mortality risk, an insight less emphasized in previous predictive frameworks. By harmonizing these determinants, our findings present a comprehensive and updated perspective on post-STEMI mortality trajectories. Moreover, while prior investigations laid the foundational groundwork, our refined insights are poised to enhance the precision of clinical decision-making.

### Explanation of methodology and findings

We employed LASSO regression, an analytical technique that selectively reduces certain regression coefficients to zero, emphasizing the most relevant predictive variables. One of the key advantages of this method is its resistance to overfitting, especially when confronted with a plethora of potential predictors. Accordingly, the five predictors we identified are arguably the most closely correlated with post-discharge mortality risks in STEMI patients.

### Novelty of findings

Our model provides a approach to estimating mortality risk in STEMI patients, blending a range of clinical, demographic, and treatment aspects. It potentially helps in identifying patients who might benefit from customized care after discharge, influencing therapeutic decisions such as medication adjustments and lifestyle considerations. We recommend adaptable steps for healthcare professionals to integrate this model into their practice, potentially enhancing patient care and outcomes.

### Study limitations

It is crucial to note that our research, being retrospective in nature, may be susceptible to selection bias. Moreover, given that our patient cohort was exclusively sourced from Xiangtan Central Hospital, caution should be exercised when extrapolating our findings to broader populations. Additionally, external validation in more diverse and larger populations is essential to confirm the applicability of our findings. Our study potentially overlooked specific covariates, such as dietary habits and physical activity of patients. Future research should be broader, incorporating multiple centers and considering a more diverse array of covariates.

### Directions for future research

In light of our findings, subsequent studies should delve deeper into the mechanistic roles of ACEI/ARB/ARNI in mitigating mortality risks for STEMI patients. A comparison of our model with other well-established models could also provide insightful results. Furthermore, it's imperative to assess the applicability and accuracy of our model across varied populations and geographical locations.

## Conclusion

In this study, a model was developed to predict the 12 and 24-month post-discharge mortality risks for STEMI patients post-PCI. Using LASSO regression, we identified five key predictors. While the model shows promise in aiding risk stratification and decision-making for post-PCI STEMI patients, its broader applicability and effectiveness require confirmation through further external and temporal validation.

### Supplementary Information

**Supplementary Material 1.**

**Supplementary Material 2.**

**Supplementary Material 3.**

**Supplementary Material 4.**

## Data Availability

The datasets generated and analyzed during the current study are not publicly available due the database owner is reluctant to make them public but are available from the corresponding author upon reasonable request.
