# Supplementary material for: Development and validation of a prognostic model for predicting post-discharge mortality risk in patients with ST-segment elevation myocardial infarction (STEMI) undergoing primary percutaneous coronary intervention (PPCI)
Source: J Cardiothorac Surg. 2024 Mar 30;19:163. doi: 10.1186/s13019-024-02665-3 (PMC10981323; doi:10.1186/s13019-024-02665-3)
Supplement: Supplementary file 1 — Supplementary Material 1. [file 13019_2024_2665_MOESM1_ESM.docx]

| **Supplementary Table 1. The single-factor Cox regression analysis of the impact of ACEI/ARB/ARNI usage on mortality in two patient groups classified based on LVEF values** | | | | |
| --- | --- | --- | --- | --- |
|  | Statistics | Death/Total | HR (CI 95%) | P-value |
| **LVEF less than 50** | | | | |
| Not taking ACEI/ARB/ARNI | 29 (10.47%) | 7/29 | reference | |
| Taking ACEI/ARB/ARNI | 248 (89.53%) | 13/248 | 0.17 (0.07, 0.44) | 0.0002 |
| **LVEF greater than or equal to 50** | | | | |
| Not taking ACEI/ARB/ARNI | 68 (17.57%) | 9/68 | reference | |
| Taking ACEI/ARB/ARNI | 319 (82.43%) | 6/319 | 0.14 (0.05, 0.38) | 0.0002 |
| **Total** | | | | |
| Not taking ACEI/ARB/ARNI | 97 (14.61%) | 16/97 | reference | |
| Taking ACEI/ARB/ARNI | 567 (85.39%) | 19/567 | 0.16 (0.08, 0.31) | <0.0001 |
