# Supplementary material for: Development and validation of a prognostic model for predicting post-discharge mortality risk in patients with ST-segment elevation myocardial infarction (STEMI) undergoing primary percutaneous coronary intervention (PPCI)
Source: J Cardiothorac Surg. 2024 Mar 30;19:163. doi: 10.1186/s13019-024-02665-3 (PMC10981323; doi:10.1186/s13019-024-02665-3)
Supplement: Supplementary file 2 — Supplementary Material 2. [file 13019_2024_2665_MOESM2_ESM.docx]

| **Supplementary Table 2: Model Evaluation Metrics on Training and Validation Sets** | | |
| --- | --- | --- |
| **datatset** | Trianset | Validationset |
| **Cutoff** | 0.087 | 0.056 |
| **AUC** | 0.88 | 0.795 |
| **AUC.SE** | 0.038 | 0.085 |
| **AUC.low** | 0.805 | 0.628 |
| **AUC.up** | 0.954 | 0.961 |
| **P-value** | <0.001 | 0.007 |
| **ACC** | 0.909 | 0.85 |
| **ACC.low** | 0.909 | 0.849 |
| **ACC.up** | 0.91 | 0.851 |
| **SEN** | 0.75 | 0.667 |
| **SEN.low** | 0.56 | 0.289 |
| **SEN.up** | 0.94 | 1 |
| **SPE** | 0.917 | 0.856 |
| **SPE.low** | 0.891 | 0.806 |
| **SPE.up** | 0.942 | 0.905 |
| **PLR** | 9 | 4.619 |
| **PLR.low** | 6.039 | 2.384 |
| **PLR.up** | 13.413 | 8.95 |
| **NLR** | 0.273 | 0.39 |
| **NLR.low** | 0.128 | 0.125 |
| **NLR.up** | 0.583 | 1.21 |
| **PPV** | 0.288 | 0.125 |
| **PPV.low** | 0.165 | 0.01 |
| **PPV.up** | 0.412 | 0.24 |
| **NPV** | 0.988 | 0.988 |
| **NPV.low** | 0.977 | 0.972 |
| **NPV.up** | 0.998 | 1.004 |
| **PPA** | 0.75 | 0.667 |
| **PPA.low** | 0.56 | 0.289 |
| **PPA.up** | 0.94 | 1.044 |
| **NPA** | 0.917 | 0.856 |
| **NPA.low** | 0.891 | 0.806 |
| **NPA.up** | 0.942 | 0.905 |
| **TPA** | 0.909 | 0.85 |
| **TPA.low** | 0.883 | 0.801 |
| **TPA.up** | 0.936 | 0.899 |
| **KAPPA** | 0.378 | 0.169 |
| **KAPPA.low** | 0.233 | 0.006 |
| **KAPPA.up** | 0.522 | 0.331 |
| Abbreviations: AUC:Area Under the Receiver Operating Characteristic Curve; SE:Standard Error; ACC:Accuracy; SEN:Sensitivity; SPE:Specificity; PLR:Positive Likelihood Ratio; NLR:Negative Likelihood Ratio; PPV:Positive Predictive Value; NPV:Negative Predictive Value; PPA:Positive Percent Agreement; NPA:Negative Percent Agreement; TPA:Total Percent Agreement; KAPPA:Kappa statistic. | | |
