# Supplementary figures and images for: Development and validation of a prognostic model for predicting post-discharge mortality risk in patients with ST-segment elevation myocardial infarction (STEMI) undergoing primary percutaneous coronary intervention (PPCI)
Source: J Cardiothorac Surg. 2024 Mar 30;19:163. doi: 10.1186/s13019-024-02665-3 (PMC10981323; doi:10.1186/s13019-024-02665-3)

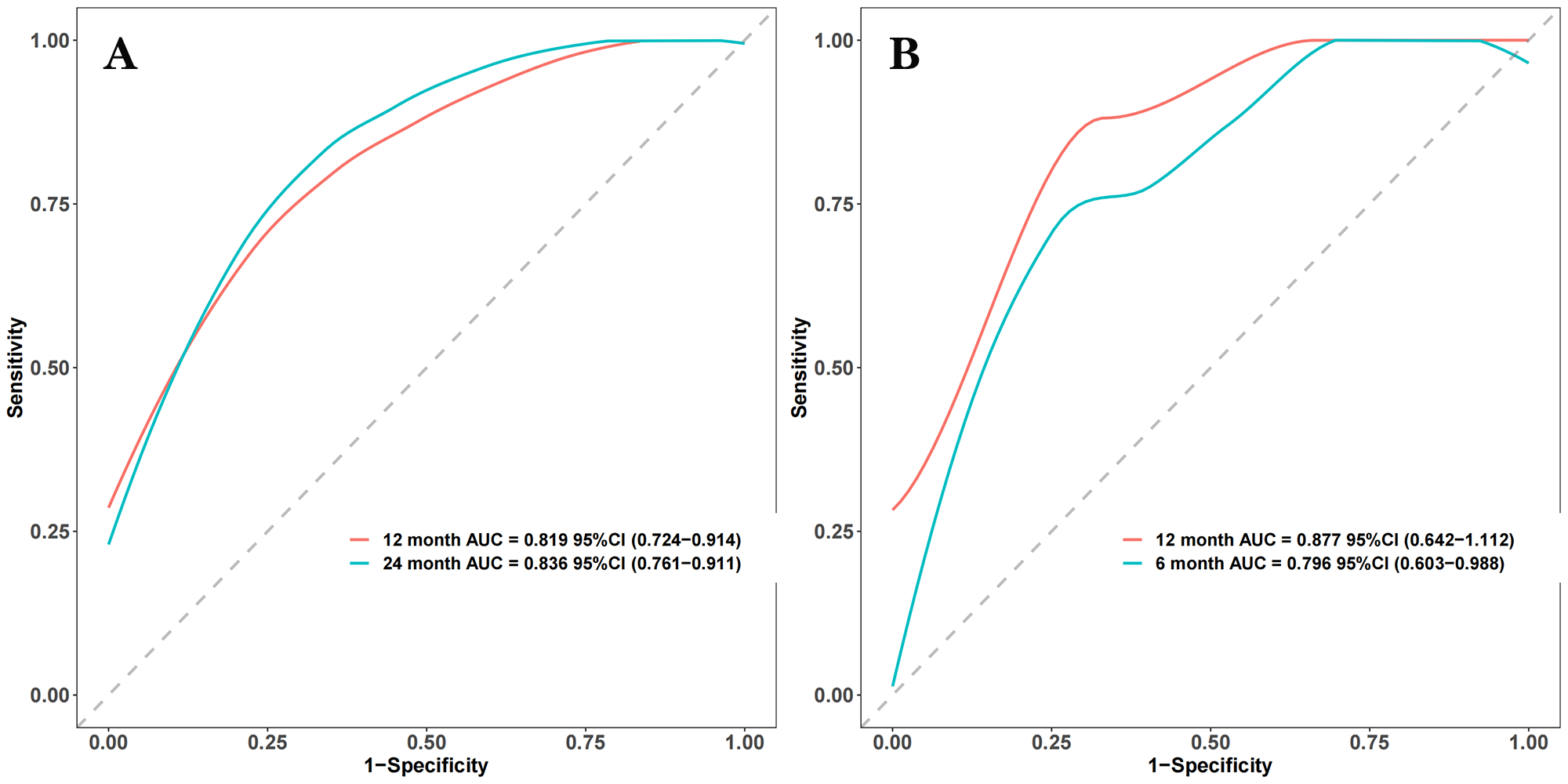

Supplement: Supplementary file 3 — Supplementary Material 3. [file 13019_2024_2665_MOESM3_ESM.tif]

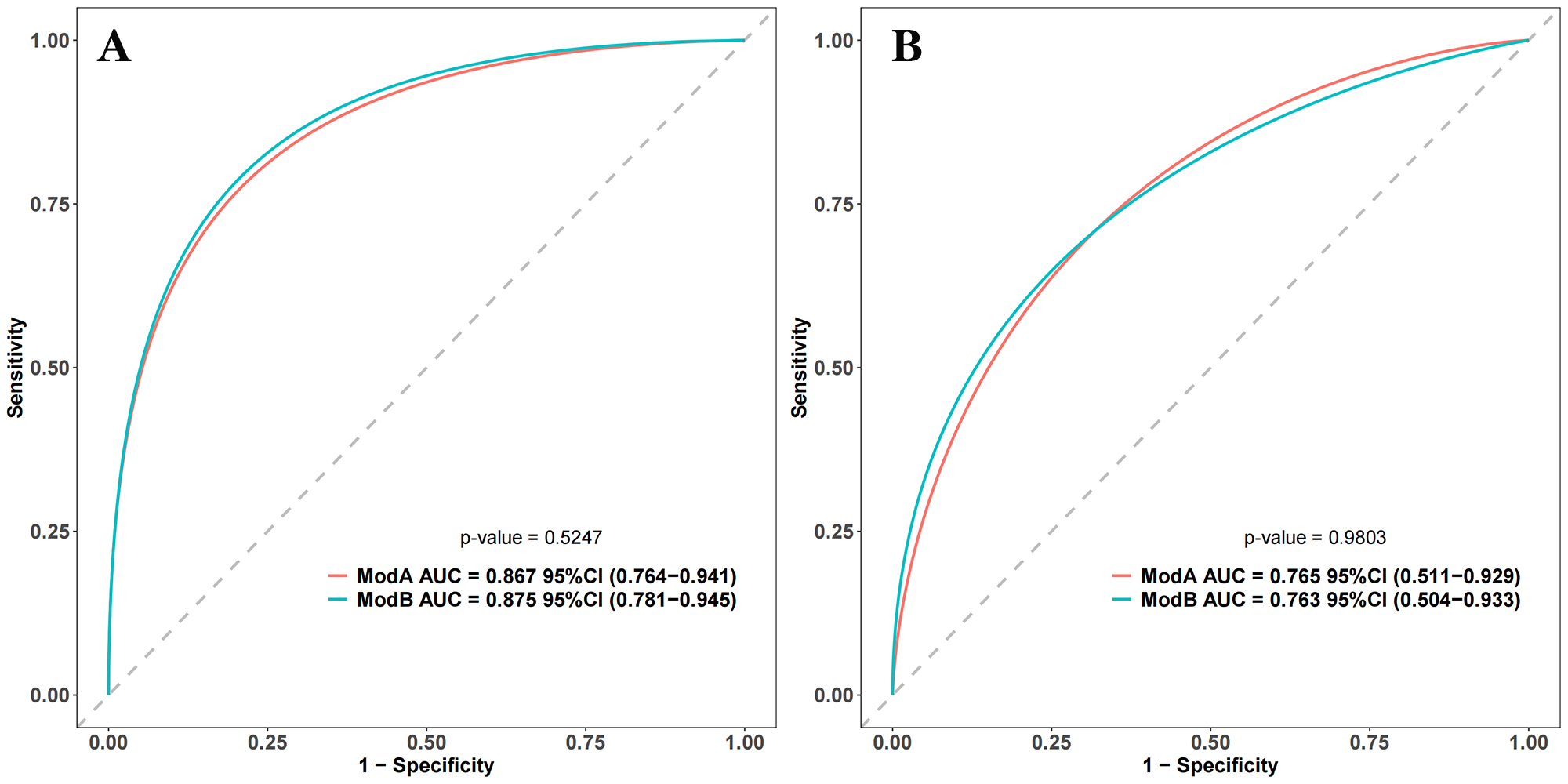

Supplement: Supplementary file 4 — Supplementary Material 4. [file 13019_2024_2665_MOESM4_ESM.tif]
